# Pulmonary Adenocarcinoma in Malignant Pleural Effusion Enriches Cancer Stem Cell Properties during Metastatic Cascade

**DOI:** 10.1371/journal.pone.0054659

**Published:** 2013-05-01

**Authors:** Su-Feng Chen, Yaoh-Shiang Lin, Shu-Wen Jao, Yun-Ching Chang, Chia-Lin Liu, Yu-Ju Lin, Shin Nieh

**Affiliations:** 1 Department of Dental Hygiene, China Medical University, Taichung, Taiwan; 2 Department of Otolaryngology, Kaohsiung Veterans General Hospital, Kaohsiung, Taiwan; 3 Division of Colon and Rectal Surgery, National Defense Medical Center and Tri-Service General Hospital, Taipei, Taiwan; 4 Graduate Institute of Life Sciences, National Defense Medical Center and Tri-Service General Hospital, Taipei, Taiwan; 5 Department of Pathology, National Defense Medical Center and Tri-Service General Hospital, Taipei, Taiwan; National Cancer Center, Japan

## Abstract

**Background:**

Metastasis occurs in a series of discrete steps involving invasion, angiogenesis, lymphovascular space permeation, and establishment of secondary tumors. Malignant pleural effusion (MPE), a type of tumor metastasis, is usually a poor prognostic sign for patients with pulmonary adenocarcinoma, although its underlying mechanism has received less attention than other types of metastases have. The objective of the current study was to confirm whether cancer stem cells (CSCs) in MPE contribute to the “metastatic cascade” through the epithelial – mesenchymal transition (EMT), anoikis, and adaptation in the microenvironment.

**Methods:**

Pulmonary tissue and corresponding cell blocks of MPE samples from 20 patients with primary adenocarcinoma were analyzed by immunohistochemical staining with CSC-representative markers (CD133, Nanog, and OCT-4) and EMT-associated markers (E-cadherin and vimentin). Correlations between these variables and clinico-pathological parameters were analyzed. Primary cultures from eight cases of MPE were investigated to characterize the CSC properties, including marker expression, sphere formation, and differentiation.

**Results:**

Expressions of CSC-representative markers for 20 cases of MPE cell blocks were quite diverse and variable ranging from 15% to 90%. Stronger expression of CSC-representative markers and alteration of EMT-associated markers were found at the invasive fronts and in MPEs compared with the expression in primary pulmonary tumor tissues. The expression of OCT-4 in MPEs significantly related to distant metastasis and stage, as well as inversely correlated with patient survival. Primary cultures confirmed the CSC properties in MPE. Five of eight cases of MPE yielded adequate cell clusters, which also showed variable expressions of CSC markers in addition to sphere formation and the ability for differentiation and metastasis.

**Conclusion:**

This pilot study offers a better understanding of the metastatic cascade. Establishing a model of MPE will provide further insight into the role of CSCs in metastasis and may explain the high therapeutic failure rates for patients with MPE.

## Introduction

Metastasis, the spread of malignant cells from a primary tumor to distant organs, is the main cause of treatment failure and death in cancer patients [1]. It usually comprises a series of discrete steps and requires the transport of malignant cells via blood and/or lymph vessels. In addition to blood and lymph vessels, body fluid is also a potential means of transportation for cancer metastasis [2]. Involvement of the serous membrane from the primary pulmonary adenocarcinoma causing initial malignant pleural effusion (MPE) usually indicates a late stage of cancer. According to the recent AJCC guidelines [3], MPE is metastatic, can cause considerable discomfort, and reflects a poor prognosis for the patient. Effusions may offer a unique in situ milieu to establish a “premetastatic niche,” and studying this milieu may help us understand how the cancer cells with stem cell-like properties in effusions contribute to the progression from individual to enriched cell clusters to advanced metastasizing lung cancer [4].

By reducing their cell–cell or cell–matrix interactions, cancer cells enter the serous cavities, where they detached from the parent tumor and become “homeless.” This triggers a phenomenon called “anoikis” [5]. The floating malignant cells in a state of anoikis in effusions are thought to succumb to apoptosis and to disappear. However, in reality, the opposite occurs, because these cells are uniquely capable of proliferating progressively despite the presumably unfavorable microenvironment. This raises the questions, “How can these cancer cells survive and proliferate to overcome the threat of anoikis?” and “What mechanism allows these cells to acquire survival signals and thus survive and proliferate in a floating tumor population in effusions that lack the normal solid-phase scaffolding?”

The presence of cancer stem cells (CSCs), mainly in solid tumors, has been proposed recently. CSCs are also called cancer-initiating cells because of their capacity for self-renewal, multilineage differentiation, and higher levels of malignancy [6]–[9]. Studies have shown that CSCs are resistant to therapy and seem to be responsible for tumor recurrence and metastasis [6], [10], [11]. Increasing evidence shows that the epithelial – mesenchymal transition (EMT) is involved in cancer progression and is closely associated with the “stemness” of cancer cells [12], [13]. MPE may serve as a reservoir for tumor cells in the pleural cavity, where they can progress through the EMT, become invasive again, and establish further metastases [14]. There is also a growing body of evidence suggesting a relationship between the EMT, the emergence of CSCs, and drug resistance [15]. MPE seems to be an ideal and unique model for studying the relationship between the EMT and CSC properties.

Several studies have suggested that suppression of anoikis and the EMT are linked [16], [17]. However, there have been few reports on the existence of CSCs in effusions. Most studies of cancer spreading have focused on the mechanisms underlying metastases via lymphatic or hematogenous routes. Less is understood about the associations between the existence and properties of CSCs, the EMT, anoikis suppression, and the tumor microenvironment in MPE. The objective of the current study was to confirm the possible role of CSCs in effusions and to explore the mechanism involved in the spread of tumors and the relationship between expression of EMT-associated markers and patient outcomes. Establishment of a model of MPE may provide a better understanding of the role of CSCs in metastasis and the high therapeutic failure rates in patients with MPE.

## Results

### Confirmation and identification of adenocarcinoma in the lung and pleural effusions

**Figure 1:**
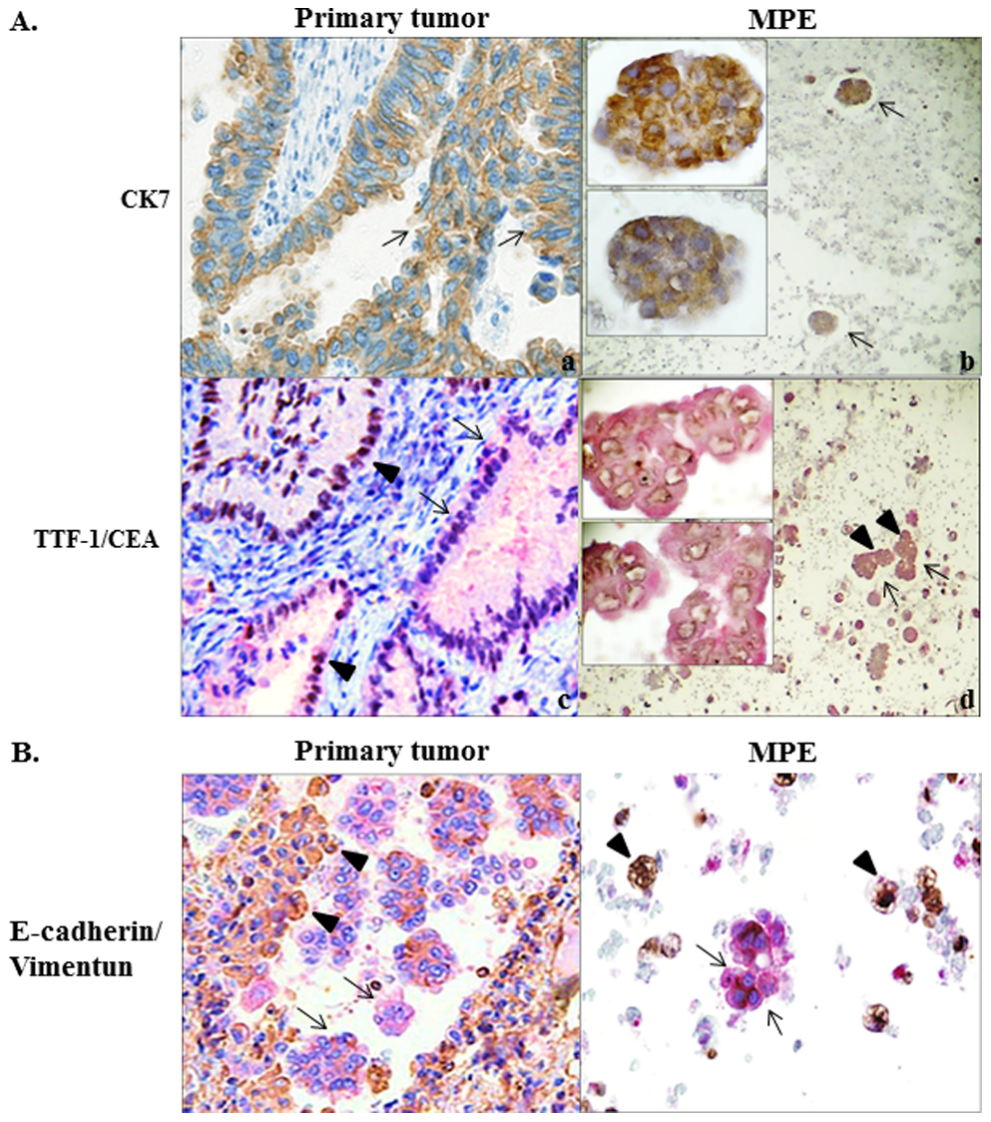
Confirmation and identification of adenocarcinoma in the lung and pleural effusions with subsequent coexpressions of EMT-associated markers. (A) Immunohistochemical identification of adenocarcinoma cells showed positive results of CK-7 (arrows) in both primary pulmonary tumor (Magnification, 200×) and cell nests in cell block of MPE at lower and higher magnifications (a, b) (Magnification, 40× and 400×); Double-staining of TTF-1/CEA revealed that TTF-1 (brown color, arrow heads) and CEA (red color, arrows) were evenly positive-stained in primary pulmonary tumor (c) (Magnification, 200×), while CEA (+) cells (arrows) are stained stronger than those of TTF-1 (+) cells (arrow heads) in cell block of MPE at lower and higher magnifications (d). (Magnification, 40× and 400×). (B) Double-staining of E-cadherin/vimentin showed that decreased E-cadherin (brown color, arrows) and increased vimentin (red color, arrow heads) expressions were found at the invasive fronts of primary pulmonary tumor and in cell block of MPE. (Magnification, 200×) (C).

Tissues from primary pulmonary adenocarcinoma and the corresponding MPE cell blocks from 20 patients were selected as study groups. Those paired study groups were subjected to histopathological and cytological identification. The immunohistochemical expression of CK-7 and TTF-1/CEA was examined. CK-7 and TTF-1 can be detected immunohistochemically in most pulmonary adenocarcinomas [18]. CEA is of value in confirming the presence of an adenocarcinoma and is considered a better alternative for differentiating between invasive malignant and nonmalignant effusions [19]. Expression of CK-7 was detected in both primary lung tumors and cell nests in MPE cell blocks. Double staining for TTF-1/CEA showed that TTF-1, whose nuclear staining appeared as a brown color, and CEA, whose cytoplasmic staining appeared as a red color, stained evenly in primary lung tumors, whereas CEA-positive cells stained stronger than TTF-1-positive cells in MPE cell blocks did (Figure 1A).

### Expressions of EMT-associated markers in primary tumors and pleural effusions

An immunohistochemical study using the EMT-associated markers E-cadherin and vimentin, showed a variety of expression patterns in the 20 pairs of primary tumor tissue samples and MPE cell blocks. The expression levels were related to the location in both the primary pulmonary adenocarcinomas and the MPE cell blocks. A higher expression of vimentin and a lower expression of E-cadherin were found in the invasive cell nests and MPE than in the center of the primary tumors, indicating that alterations of the EMT markers were associated with local invasion and malignant involvement in the pleural cavity (Figure 1B).

### Characterization and distribution of CSC-representative markers related to the microenvironment

**Figure 2:**
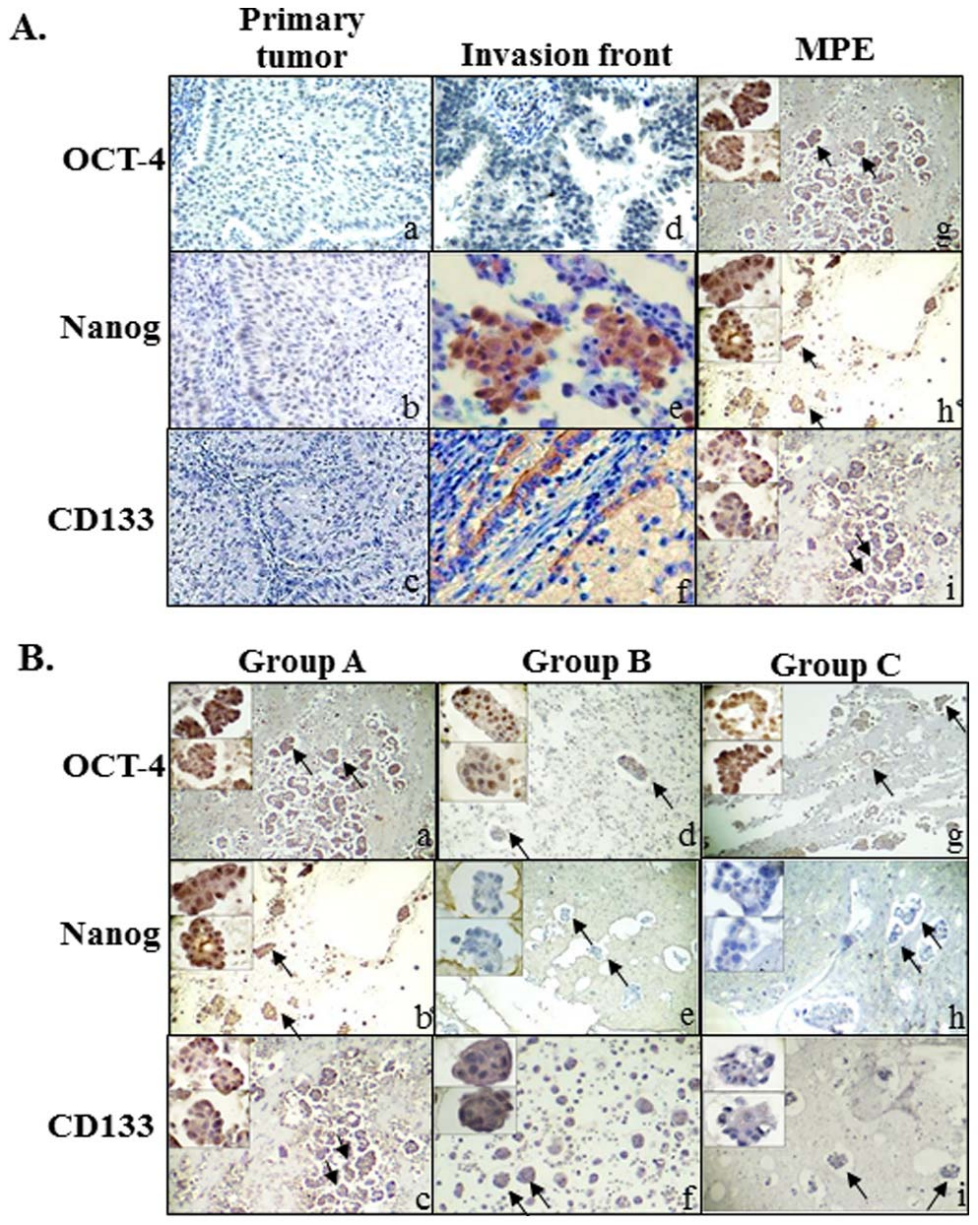
Characterization and distribution of CSC-representative markers related to the microenvironment. (A) Immunohistochemical results showed immunoreactivity of three CSC-representative markers (OCT-4, Nanog, and CD133) in the center of the primary tumor (a, b & c) (Magnification, 200×). Stronger immunoreactivity was shown at the invasive fronts (d, e & f) (Magnification, 200×) and many cancer cell clusters (arrows) in MPE at lower and higher magnifications (g, h & i). (Magnification, 40× and 400×). (**B**) Immunocytochemical characterization of cancer cell clusters in MPE showed three major combination groups (positive OCT-4, combined with positive or negative Nanog, and CD133 expressions) were identified: Group A, positive for all three CSC-representative markers (a, b &c); Group B, positive for OCT-4 and CD133, but negative for Nanog (d, e & f); Group C, positive for OCT-4, but negative for Nanog and CD133 (g, h & i) at lower and higher magnifications. (Magnification, 40× and 400×).

Most of the MPE samples showed variable immunoreactivity to CD133, Nanog, and OCT-4, and combinations of staining elicited different intensities and distributions. A relatively weak expression of OCT-4, Nanog, and CD133 was always found in the center of the primary pulmonary tumors. Stronger expression levels of OCT-4, Nanog, and CD133 were detected at the invasive front, located at the interface between the tumor cell nests and stromal tissue in primary pulmonary adenocarcinomas, and at the outer margins of the tumor cell nests in the MPE cell blocks (Figure 2A). Regardless of intensity expressed in each case, all of the 20 cases of MPE were more or less immunoreactive for one or two or all of three- CSC-representative markers. In other words, there was no single case of MPE negative for all three- CSC-representative markers. Because OCT-4 was regarded as the most important CSC-representative marker, the percentage related to OCT-4 expression present in 20 cases of MPE cell blocks were quite diverse ranging from 10% to 90%. Due to intratumoral heterogeneity, three different combination patterns of positive OCT-4 combined with positive or negative Nanog, and CD133 expressions were identified in the MPEs (Figure 2B). Four groups were classified including three major positive OCT-4 groups and one negative OCT-4 group. Three of 20 cases of MPE were positive for all three markers in group A (3/20, 15%). Five of 20 cases of MPE were positive for OCT-4 and CD133, but were negative for Nanog in group B (5/20, 25%). Ten of 20 cases of MPE were positive for OCT-4 but negative for Nanog and CD133 in group C (10/20, 50%). The two remaining cases classified as group 4 were negative for OCT-4 and Nanog, but positive for CD133 (2/20, 10%). The original data and statistical analysis of 20 cases of MPE were presented in Table S1, Tables 1 and 2.

**Table 1:** Comparative analysis of the results of immunoexpression of three CSC-representative markers showing variable positive and negative numbers, and proportions in 20 patients with MPE.

	Case Number (%)		
	OCT-4	Nanog	CD133
Positive	**18 (90%)**	**6 (30%)**	**10 (50%)**
Negative	**2 (10%)**	**14 (70%)**	**10 (50%)**

**Table 2:** Classification of immunoexpressions of three CSC-representative markers and their combinations into four groups showing variable corresponding numbers and proportions in 20 patients with MPE.

	Group A	Group B	Group C	Group D
**Case Number (%)**	**3 (15%)**	**5 (25%)**	**10 (50%)**	**2 (10%)**

Group A: positive for all three CSC-representative markers
Group B: positive for OCT-4 and CD133, but negative for Nanog
Group C: positive for OCT-4, but negative for Nanog and CD133
Group D: positive for CD133 but negative for OCT-4 and Nanog

### Correlation of immunoexpression of CSC-representative markers with clinico-pathological parameters

**Figure 3:**
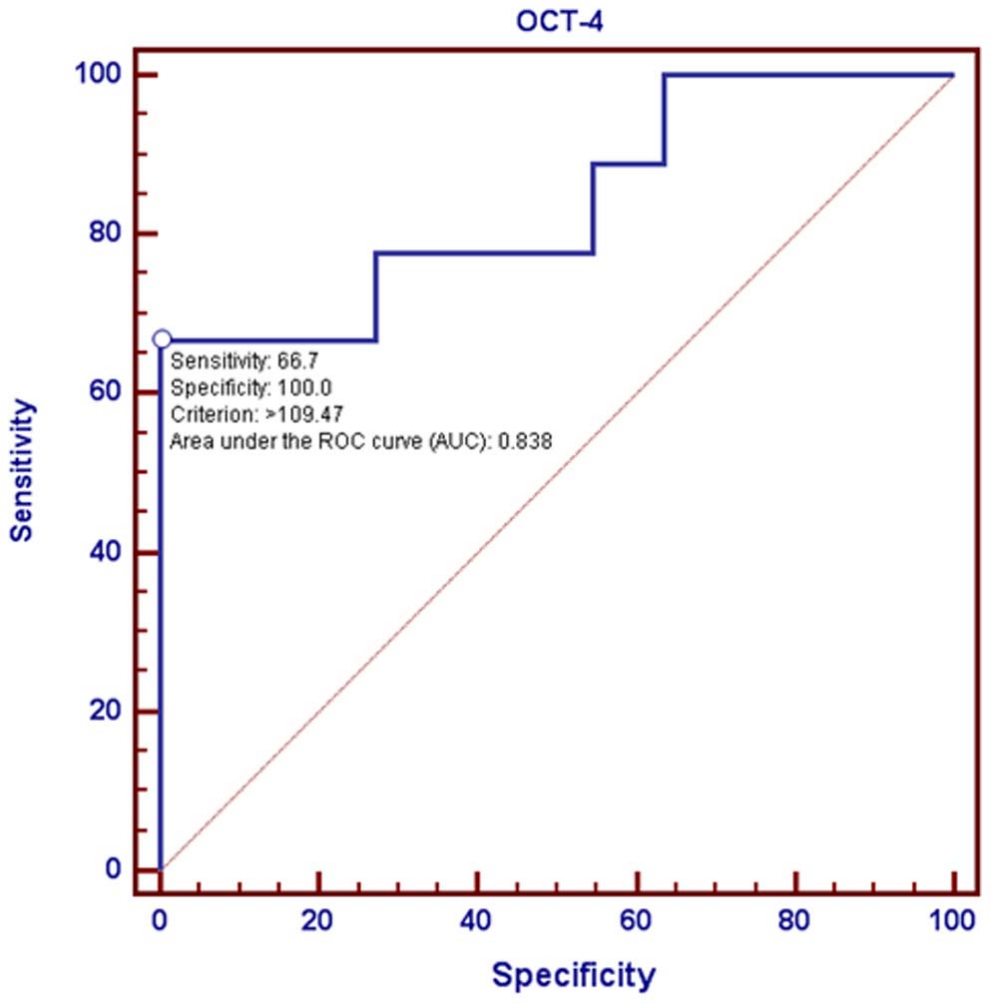
Correlation of immunoexpression of OCT-4 with metastasis. Receiver Operating Characteristic Curve (ROC) using the percentage of OCT-4 as a predictor of metastasis demonstrated a relatively high sensitivity (66.7%) and an absolute specificity (100%). The area under ROC Curve was 83.8%.

**Figure 4:**
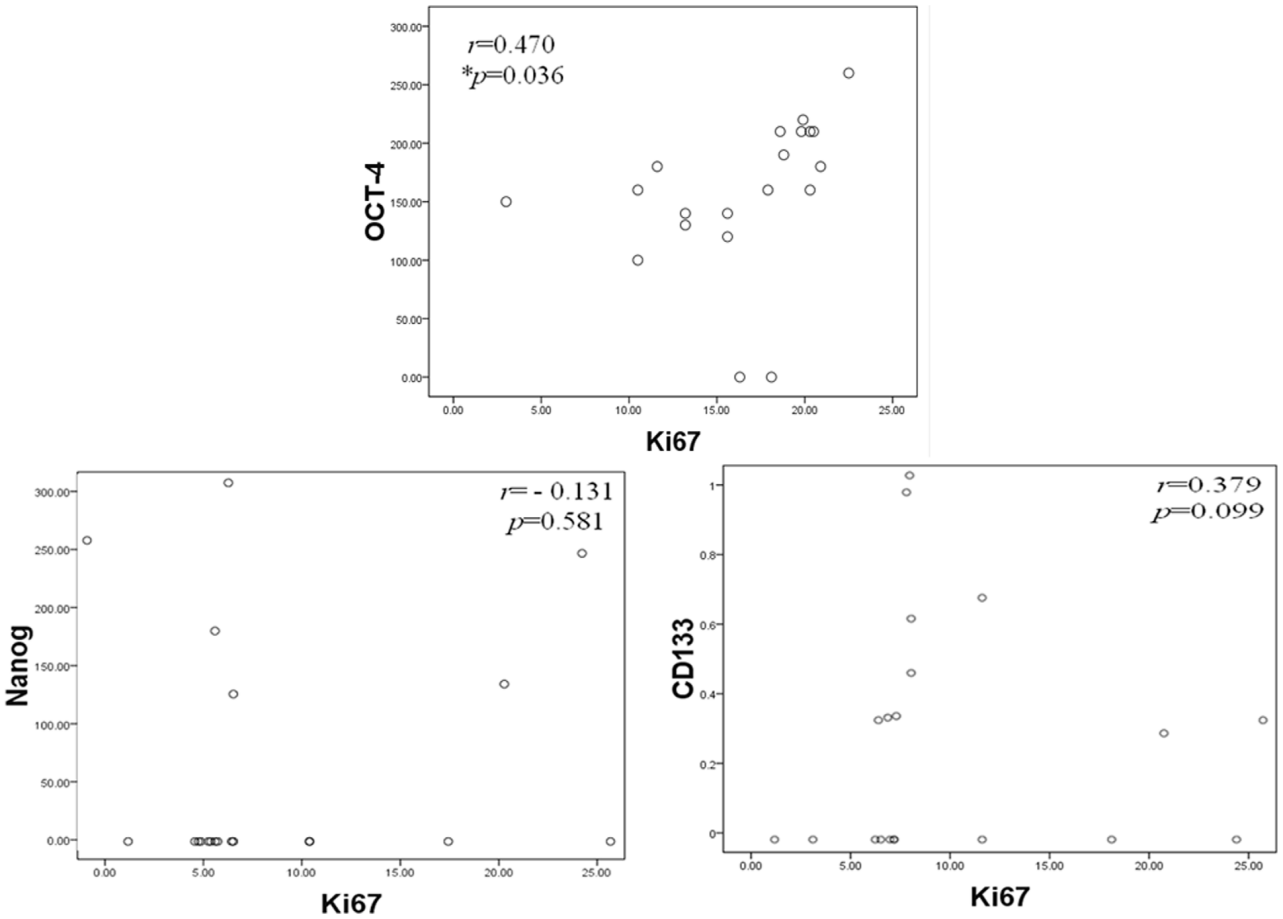
Correlation of immunoexpression of CSC-representative markers with Ki67. Comparative analysis using linear regression test of CSC-representative markers (Nanog, OCT-4, and CD133) with Ki67 showed that only OCT-4 was found positively correlated with Ki67. However, there were no correlations at all between the expressions of Nanog and CD133 and Ki67.

**Figure 5:**
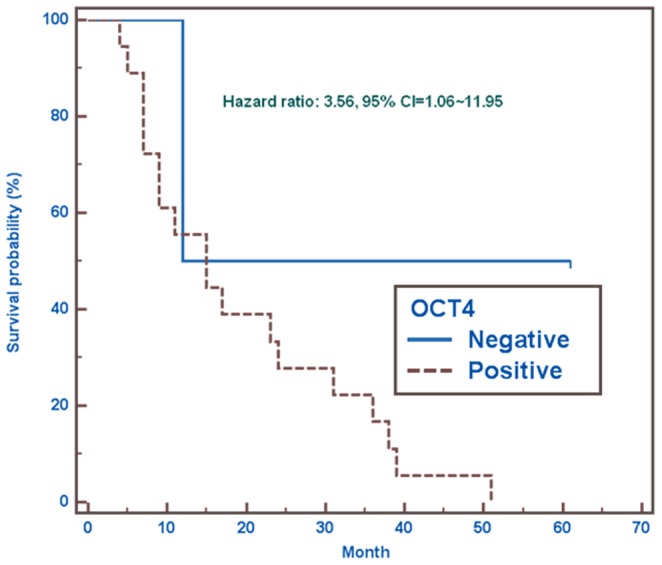
Correlation of immunoexpression of OCT-4 with survival. The Kaplan-Meier survival analysis after a 60-month follow-up showed that the estimated risk of the event of interest occurring in OCT-4 positive group was 256% higher than in OCT-4 negative group (Hazard ratio = 3.56, 95% CI = 1.06∼11.95).

Comparative analysis between CSC-representative markers using immunoreactivity score calculation system and clinico-pathological parameters demonstrated that there was a statistical significance between the expression of OCT-4 and distant metastasis (*p* = 0.0121). There was also a statistical significance between the expression of OCT-4 and stage (*p*<0.0001). However, there was no statistical significance between the expression of OCT-4 and other clinico-pathological parameters. No statistical significance was found between the expression of Nanog and CD133 and all clinico-pathological parameters. Following the adjustment of clinico-pathological parameters, OCT-4 was also found positively correlated with increasing odds ratio related to metastasis (Tables 3, 4 and 5). Using the percentage of OCT-4 as a predictor of metastasis, such measurement was quite sensitive with high specificity (Figure 3). Further linear regression analysis showed that immunoexpressions of OCT-4 were positively correlated with Ki67 (*r* = 0.470, *p* = 0.036), nevertheless there were no correlations of Nanog and CD133 with Ki67 (Figure 4). The expressions of OCT-4 were also statistically and inversely correlated with patient survival following a 60-month follow-up using the Kaplan-Meier survival analysis (Figure 5).

**Table 3:** Univariate analysis for correlation between metastasis and immunoexpression of three CSC-representative markers, age and Ki67 in 20 patients with MPE.

Continuous Variable	Metastasis								p-value
	No (n = 11)				Yes (n = 9)				
	Sum of Scores	Expected Under H0	Std Dev Under H0	Mean Score	Sum of Scores	Expected Under H0	Std Dev Under H0	Mean Score	
**OCT-4**	82	115.5	13.16	7.45	128	94.5	13.16	14.22	**0.0121**
**Nanog**	101.5	115.5	10.68	9.23	108.5	94.5	10.68	12.06	0.2061
**CD133**	110.5	115.5	12.29	10.05	99.5	94.5	12.29	11.06	0.7143
**Age**	130	115.5	13.14	11.82	80	94.5	13.14	8.89	0.2868
**Ki67**	135	115.5	13.16	12.27	75	94.5	13.16	8.33	0.1489

Wilcoxon rank sums test.

**Table 4:** Univariate analysis for correlation between metastasis and clinico-pathological variables in 20 patients with MPE.

Categorical Variable		Metastasis		p-value
		No (n = 11)	Yes (n = 9)	
**Age** **group**	<65	4 (36.36)	6 (66.67)	0.3698
	≧65	7 (63.64)	3 (33.33)	
**Gender**	Male	5 (45.45)	5 (55.56)	1.000
	Female	6 (54.55)	4 (44.44)	
**T Classification**	T3	3 (27.27)	0 (0)	0.2184
	T4	8 (72.73)	9 (100)	
**N Classification**	N0	2 (18.18)	2 (22.22)	1.000
	N1∼3	9 (81.82)	7 (77.78)	
**Stage**	III	10 (90.91)	0 (0)	**<0.0001**
	IV	1 (9.09)	9 (100)	

Chi-square test with Fisher's exact test.

**Table 5:** Multiple logistic regression analysis for risk factor in terms of metastasis.

Variable	Coefficient	Std. Error	Odds ratio	95% CI	*p*- value
OCT-4	0.0454	0.0217	1.0464	1.0029 ∼1.0918	0.0361

### Different presentation with characterization of enriched cell clusters from MPE using both nonadhesive and conventional culture systems

**Figure 6:**
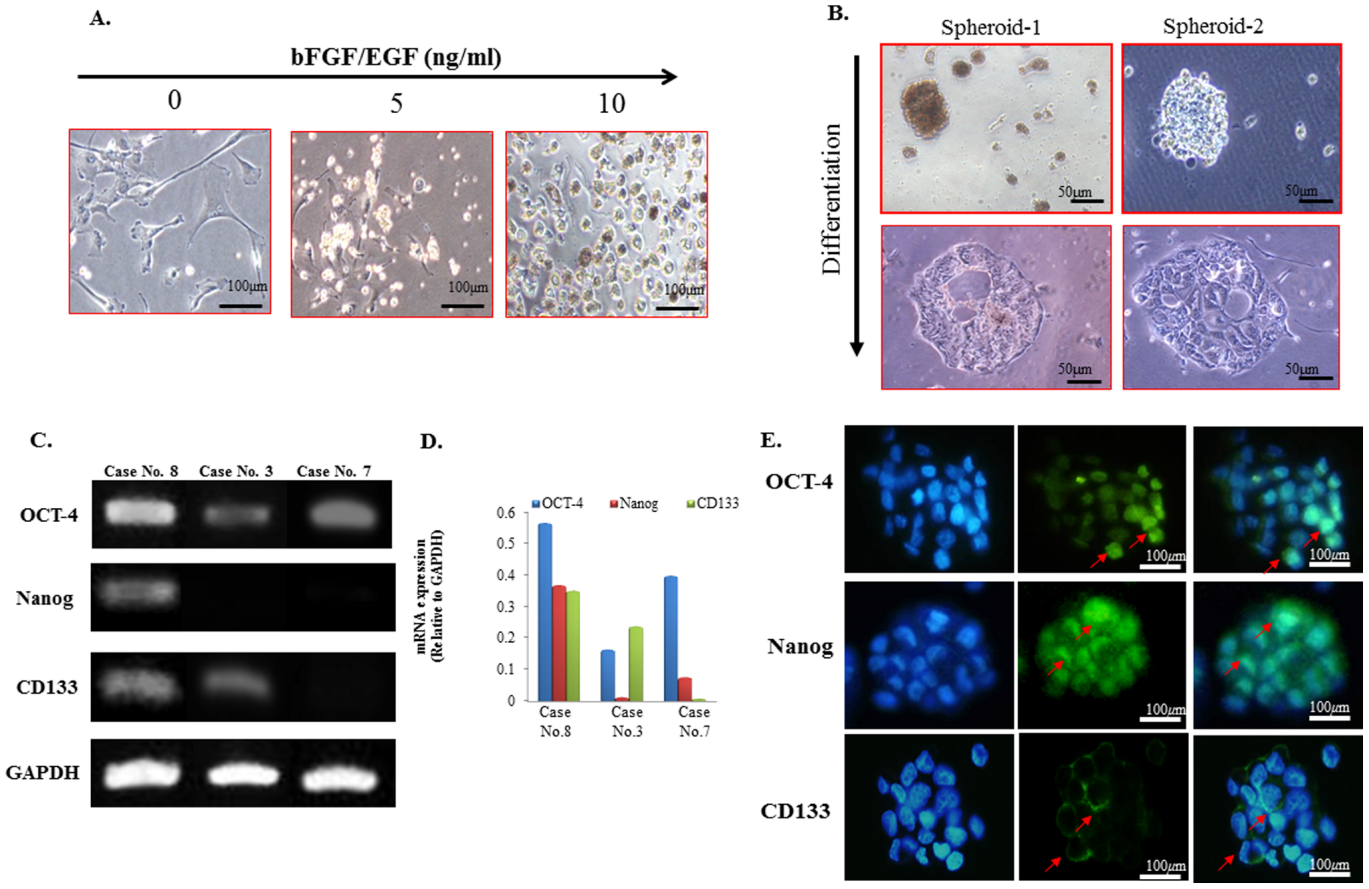
Characterization of spheroids from MPE for CSC properties in primary cell cultures. (A) Phase-contrast photomicrographs of primary culture of MPE from patients with primary pulmonary adenocarcinoma using serum-free medium supplemented with increasing doses of bFGF and EGF showed a gradual morphological alteration of adenocarcinoma cells from adherence to the plate turning into small movable clusters and finally becoming into the spheroid forms. (Left to right panel, magnification, 200×). (B) Differentiation induction of two representative cases demonstrated a reversed, morphological alteration turning the spheroid in MPE into an original glandular architecture with luminal channels. (C) A RT-PCR analysis of spheroids in three representative cases (Case No. 8, 3 and 7) of MPE showed the expression of the OCT-4, and Nanog and CD133 genes was variably upregulated in there representative cases in patients with MPE. (D) Corresponding quantitative data demonstrated the comparisons among the expression of the OCT-4, and Nanog and CD133. (E) Immunofluorescence analysis of CSC-representative markers of spheroids demonstrated positive expressions of OCT-4, Nanog, and CD133, as indicated by the arrows (Magnification, 200×).

Eight cases of MPE were prospectively collected for the primary culture and their clinico-pathological data were shown in Table 6. The primary culture cells from MPE using nonadhesive culture system described in our previous report [20] displayed three-dimensional (3-D) clusters with a grape-like morphology (Figure S1). The doubling time duration of cell culture, and morphology observed varied considerably between samples. However, primary culture by conventional culture system using serum-free medium supplemented with increasing doses of bFGF and EGF disclosed a gradual morphological alteration. The cultured cancer cells initially adhered to the plate, then dissociated and detached from the plate, and finally transformed into small, movable, spheroid clusters (spheroids) displaying a 3-D and/or grape-like morphology (Figure 6A).

**Table 6:** Clinical data and cytological diagnosis of eight MPE specimens.

Case No.	age/sex	specimen source	diagnosis
1	59/M	pleural fluid	adenocarcinoma of lung with metastases to lymph nodes, bone and liver cT4N3M1b, stage IV
2	74/M	pleural fluid	adenocarcinoma of lung with metastases to brain and bone, cT4NxM1, stage IV
3	59/M	pleural fluid	adenocarcinoma of lung with metastases to lymph nodes and brain, cT4N3M1b, stage IV
4	88/M	pleural fluid	adenocarcinoma of lung, with lung to lung, lymph nodes, bone and suspected brain metastases, cT4N2M1b, stage IV
5	82/F	pleural fluid	adenocarcinoma of lung with left pleura metastasis, cT4NxM1a, stage IV
6	58/M	pleural fluid	Adenocarcinoma of lung, with lung to lung metastasis, cT4N0M0, stage IIIB
7	65/M	pleural fluid	adenocarcinoma of lung with lymph nodes and left pleura metastases, cT2N2M1b, stage IV
8	78/F	pleural fluid	adenocarcinoma of lung with right pleura metastasis, cT4NxM1a, stage IV

### Characterization of spheroids for CSC properties including sphere formation, and differentiation in MPE primary cell cultures

Samples from five of eight (62.5%) MPE patients using nonadhesive culture system yielded enriched sphere formation after culture for 7 days (Table 7). We subsequently recultured these spheroids in conventional adhesive culture dishes for differentiation induction. The spheroids consisting of putative CSCs exhibited the ability of differentiation reversing the above process turning the 3-D cell clusters into two-dimensional structures with a glandular morphology (Figure 6B). Immunofluorescence analysis with morphological identification of CSC-representative markers of spheroids demonstrated the presence of expressions of OCT-4, Nanog, and CD133 and further RT-PCR analysis showed that variable upregulation of OCT-4, and Nanog and CD133 gene expression in three representative cases. Three different patterns of expression were observed for OCT-4, Nanog, and CD133 (Figures 6C, D and E). Seven of eight (87.5%) primary culture cell clusters of MPE were positive for the CSC-representative markers, and only one (12.5%) was negative for all three CSC-representative markers. Stronger expressions of OCT-4 were found quantitatively than those of Nanog and CD133 in all three representative cases. These data agreed with those obtained from the cell blocks.

**Table 7:** The ability of sphere formation of primary culture cells from MPE.

Case No.	age/sex	Sphere formation
1	59/M	Yes
2	74/M	No
3	59/M	Yes
4	88/M	No
5	82/F	Yes
6	58/M	No
7	65/M	Yes
8	78/F	Yes

## Discussion

Metastasis usually occurs in a series of discrete steps that can be modeled into a metastatic cascade [21]. However, the mechanism underlying the tumor involvement in the metastatic cascade, resulting in the malignant effusion in serous cavities, has received little attention and therefore attracted our research interest. In this study, we reconfirmed the characteristic immunohistochemical and immunocytochemical presentations of each paired group of samples from the same patient, showing the variable intensities and distributions of CK-7 and TTF-1/CEA expression in primary lung adenocarcinoma tissues and cancer cell nests in cell blocks from the effusion. Some of the samples were strongly immunoreactive for the identifying markers and some presented irregular immunoreactivity. This may indicate intratumoral heterogeneity and may explain the potential therapeutic resistance, as well as the capacity for early invasion and metastasis to the pleural cavity. After the early events of the EMT were initiated, including the loss of adhesion and anchorage, the increased mobility, and the morphological transformation, the primary lung cancer cells entered the pleural cavity, leading to MPE [22]. Our study also demonstrated qualitative discrepancies in the expression levels of EMT-associated markers between the primary sites and the metastatic lesions. The immunoreactivity of the representative EMT-associated markers, E-cadherin and vimentin, varied between the primary lung tumor tissues and the cancer cell nests in the effusions, indicating the presence of the EMT phenomenon in the primary lung tumors and MPEs during tumor progression. Furthermore, the expression of vimentin tended to be stronger and the expression of E-cadherin weaker at the invasive front, the interface between the tumor and stromal tissues, than in the center of the tumor proper.

During invasion, and soon after the tumor cells lose contact with the basement membrane, they encounter another barrier to metastasis, anoikis [5], [23]. The detached tumor cells, individually or in small clusters, are uniquely capable of proliferating and growing, despite their presumably unfavorable locale [24]. The effusions offer a premetastatic niche, which functions as a dynamic reservoir that can supply the cancer tissue through autocrine and/or paracrine interactions with growth factors. Such growth factors include vascular endothelial growth factor, basic fibroblast growth factor, and transforming growth factor β and other stimuli, such as chemokines and cytokines (interleukins), produced mainly by cellular components, including mesothelial cells, some stromal cells, and inflammatory cells [25]–[28]. As the effusions accumulate, the microenvironment appears crucial for allowing the cancer cells to interact sufficiently with their surroundings, through perfusion rather than through the anatomical architecture of the blood flow [29]. When cancer cells become involved in such distinct microdomains, they soon orchestrate the surrounding stromal cells and alter their phenotypes to facilitate cancer progression. Therefore, the microenvironment of the effusions may accelerate the tumor's aggressive potential by dynamically triggering the properties of CSCs *in situ*. The factors or cytokines in the effusion that are involved in signaling or interacting with the tumor cells and how they cross-talk with each other to in order to provoke the potential of the CSCs in the MPE are worthy of further investigation.

Although the “seed and soil hypothesis” has long been challenged, it explains how effusions provide a unique microenvironment that can be regarded as the fertile “soil”, in a liquid state, waiting to be “seeded” with tumor cells [30], [31]. As seen in ours and other studies, effusions are unique microenvironments that not only keep cancer cells alive, but also further their invasion and promote their growth. The rapid tumor cell growth and metastasis that occur in effusions may be attributable to the presence of a small subpopulation of cancer cells, the CSCs, that display many EMT properties [31], [32]. Increasing evidence suggests that there is a link between CSCs and the EMT. If so, certain oncogenes must be involved in the stemness of tumor cells with invasive capability and their potential to self-renew [12], [13], [33]. The ability to survive and proliferate requires an appropriate niche, such as an effusion, which is crucial for disseminated tumor cells to establish themselves and spread as a metastasis. To our knowledge, only a few studies have reported the existence of CSCs in effusions. There is not yet a specific and simple marker for the properties of CSCs in effusions. Our data clearly demonstrate that the rates of immunoreactivity for the three CSC-representative markers (CD133, Nanog, and OCT-4) varied, with a relatively high rate of OCT-4 immunoreactivity. These three CSC-related markers were chosen because of their representative evidence. Surface CD133, also known as prominin-1, is a cell-surface transmembrane glycoprotein that has been used in the identification of putative CSCs in several malignant tumors, because they have shown increased tumorigenic potential in transplantation studies *in vitro* and *in vivo* [34]–[36], as well as an association with poor prognoses and distant metastases [37], [38]. However, the exact function of CD133 has not yet been established and requires clarification. OCT-4 and Nanog are both transcription factors essential for normal pluripotent cell development. Nanog is chiefly responsible for differentiation during embryogenesis [39], whereas OCT-4 may have long-term influences on both tissue proliferation and differentiation [40]. OCT-4 is especially expressed in embryonic stem cells and germ cells, and has been detected in specific types of testicular germ-cell tumors [40], [41]. OCT-4 is also preferentially expressed in undifferentiated human ESCs, pancreatic islets, and diffuse-type gastric cancers [42]. Therefore, it has been suggested that OCT-4 contributes to maintaining stem cell properties. However, several recent reports have suggested that as many as 25% of the cancer cells within certain tumors have the properties of CSCs [43]. Based on our data, we have estimated that the rates of immunoreactivity for these three CSC-representative markers (CD133, Nanog, and OCT-4) range from 15% to 90%, and variations in and combinations of their expression were also noted. These rates exceed the average occurrence of CSCs (25%) within certain tumors, especially solid tumors. Our results demonstrate that plenty of satisfactory putative CSCs can be isolated from MPEs with effective methods. Therefore, MPEs can be used as a better study model for stem cell research than tissue samples from certain cancers. The variable expression of CSC-specific markers might also be modulated by different microenvironments, and the strongest immunologically detectable expression of these three CSC-representative markers was found at the invasive fronts, as was seen for EMT-associated markers. Our study using linear regression analysis has demonstrated that only the expression of OCT-4 positively correlated with Ki67 (*r* = 0.470, *p* = 0.036), an excellent marker to determine the growth fraction of a given cell population and often in correlation with the prognostic value for survival and tumor recurrence (Figure 5) [44], [45]. Furthermore, the expression of OCT-4 also significantly related to distant metastasis (*p* = 0.0121) and stage (*p*<0.0001). The Kaplan-Meier analysis demonstrated the expression of OCT-4 was inversely associated with survival in patients with MPE after a long term follow-up.

In terms of these three CSC-representative markers, CSCs that expressed OCT-4 rather than CD133 or Nanog appeared to play a crucial role in their behavior in MPEs. Comparative analyses also revealed discrepancies in the immunoreactivity for these three CSC-representative markers between primary pulmonary tumors and cancer cell nests from the cell blocks in effusions. A reasonable explanation is that the CSCs in both primary pulmonary tumors and effusions can be dormant or stationary for a period of time before they become advanced invasive or metastatic. Indeed, the expression of stem cell properties varied with the microenvironment (the “stem cell niche”) in which they occurred [46], [47]. Until now, the link between CSCs and metastasis has been based on a conjectured correlation, because it does not seem a coincidence that relatively fewer metastasis-forming cells corresponded to equally fewer CSCs. These cells have the potential to form a full-blown secondary tumor, with a histological architecture and heterogeneity similar to those of the primary tumor [48]. Primary pulmonary tumors are predominantly comprised of stationary CSCs, which usually remain in a state of dormancy or inactivity. However, when the tumor spreads to the pleural cavity in an appropriate niche-producing MPE, these more immunoreactive or functioning “migratory CSCs” or “metastatic CSCs”, presenting either as small clusters or as detached larger groups of cells at the invasive fronts, not only survive but also self-proliferate to some extent. They might then invade the adjacent tissues and vessels, and prepare to migrate to the distal organs, where they will develop as another metastasis [32], [49], resulting in a more advanced and uncontrollable cancerous state Figure S2).

In addition to the influence of the intratumoral heterogeneity of the tumor proper and the composite components of the humoral and cellular factors in the MPE, some other difficulties and limitations are encountered when isolating cancer cells from the MPE. However, we successfully isolated tumor spheroids/clusters using both conventional and nonadhesive culture systems. These 3D ball-like structures extracted from the patients were obtained with some difficulty but were well preserved in primary culture, as has been reported in other studies [50], [51]. Further immunofluorescence analyses demonstrated that these spheroids/clusters expressed the properties of CSCs, staining positively for the three CSC-representative markers (Figure 6E), similar to the results for the cell blocks. Samples from five of eight (62.5%) patients with MPE yielded enriched sphere formation after culture in a nonadhesive culture system (Figures S1 and 6A). However, no spheres could be generated from the samples from three patients, probably reflecting individual differences and the complex interference of the microenvironments of effusions, in addition to patient-specific factors, such as the genetic status of EGFR, which can affect the choice of chemotherapeutic treatments.

In conclusion, the current study supports our perspective on the metastatic cascade. The platform of pleural effusions may provide a premetastatic niche for primary pulmonary tumors before their malignant involvement. MPEs are filled with tumor cells that have the properties of CSCs after their interactions with the microenvironment. To our knowledge, we have for the first time established a model of the MPE, from which may provide an insight into the roles of CSCs in metastasis and in high therapeutic failure rates. This may also offer a new direction for the preclinical development of rational therapeutics.

## Materials and Methods

### Clinical samples collection

Tissue specimens of 56 pulmonary adenocarcinoma patients with MPE were collected and retrieved from the archives of the Department of Pathology, Tri-Service General Hospital, Taipei, Taiwan from 2004 to 2009. 17 cases were either lost to follow-up or had insufficient clinico-pathological data for analysis and were therefore excluded. Among 39 paraffin blocks with MPE, there were 20 cases in pairs containing sufficient fluid prepared for satisfactory smears and cell blocks which were chosen to be the main study material. Cases of pleural biopsied specimens were not included when the pleural cytology was non-diagnostic for a suspected MPE. Follow-up work of all the study cases was conducted for at least 5 years.

### Double-staining of immunohistochemistry

After de-paraffinization and dehydration, specimens were brought to a boil in Antigen Retrieval Solution (Epitomics TandemTM IHC Staining and Detection Kit) for antigen retrieval. Wash slides with TBST for 5 min on a shaker (50 nM Tris, pH7.6, 150 nM NaCl, 0.1% Tween-20). Inactivate endogenous peroxidase by covering tissue with the peroxide block solution for 10 min then Block slides with the blocking solution for 1 hour. Dilute mouse monoclonal and rabbit monoclonal primary antibodies in primary antibody diluent to half the recommended dilution. Apply primary antibodies to each tissue section and incubate overnight in the humidified chamber (4°C). The detailed information of chosen primary antibodies is described in Table S2. Wash slides three times with TBST and incubate each section with secondary HRP-conjugated anti-mouse antibody and AP-conjugated anti-rabbit antibody for 45 minutes at room temperature. Wash slides three times with TBST and incubate freshly prepared Fast Red solution to the sections for 20 minutes at room temperature. Wash slides three times with TBST and Incubate tissue sections with freshly prepared DAB substrate to at room temperature until suitable staining develops. Rinse sections with water and counterstain with hematoxylin.

### Immunohistochemistry

In this study, the immunohistochemistry analysis was performed using an EnVision Dual Link System-HRP (DAB+) kit. After deparaffinization and dehydration, specimens were brought to a boil in 10 mM sodium citrate buffer (pH 6.0) for antigen retrieval. Specimens were then treated with 3% H2O2 and blocking solution. Wash slides three times with TBST and then incubated with primary antibody. The detailed information of chosen primary antibodies is described in supplementary information (Table S2). After being washed three times with TBST, the immunoreactivity was detected using the labeled polymer-HRP reagent (horseradish peroxides labeled polymer conjugated to goat anti-rabbit and goat-anti-mouse secondary antibody) and visualized with DAB. All the immunohistochemical sections were counterstained with Mayer's hematoxylin, dehydrated in graded concentrations of ethanol (70%, 90%, and 100%), and cover slipped routinely using permanent mounting medium.

### Evaluation of immunohistochemistry

A section subject to immunohistochemistry without the primary antibody and a cell block of pleural effusion with lymphocytosis associated with tuberculosis were used as negative controls. The human retina for CD-133, human germ cell tissues taken from a case of immature teratoma with immunoreactivities to Nanog and OCT-4 were used as positive controls. The intensities of the immunoreactivity of tumor cells were scored into three standardized categories: 0 (no staining), 1 (weak staining), 2 (moderate staining) and 3 (strongest intensity). The distributions of positive labeling were also measured as a percentage according to the percentage of positively stained tumor cells (from 0 to 100) in total tumor volume in each section. A value of 10% or higher for positively stained tumor cells was considered a positive reaction. The proportion of the positive cells to the total cancer cells in MPE was calculated under high-power magnification for each case. To compare the expressions for each case, the percent of positive cells at each level of intensity was multiplied by the corresponding intensity (from 0 to 3) to obtain an immunoreactivity score ranging from 0 to 300 as described in our previous report [52].

### Processing of malignant pleural effusion and primary culture

Eight cases suitable for requirement of primary culture with MPE were prospectively collected and their clinico-pathological data were shown in Table 6. Primary culture of MPE using nonadhesive culture systems was firstly performed. Conventional culture system for primary culture of MPE was also conducted in the meantime. Effusion specimens were extracted from the pleural cavity in patients with pulmonary adenocarcinoma. Samples were prepared by centrifugation (1500 g, 5 minutes, 4°C) and PBS washed twice. Cell pellets were suspended in a Percoll density gradient. Separation of cells was seen in three different gradient of Percoll. Bottom layer used 1.080 density Percoll, the middle used 1.050 density Percoll and the top used 1.030 density Percoll. Samples were processed by centrifugation (2400 g, 20 minutes, 4°C) and finally isolated as the white ring layer [53]. The primary culture cells were grown in RPMI1640 medium with 10% FBS and 2.5% penicillin/streptomycin in a humidified 5% CO_2_ incubator at 37°C. To avoid long periods of cell culture, which may lead to the selection of subpopulation of cells with particular growth advantages, all samples were banked in and all further studies were conducted with p4 to p6 cells.

### Reverse transcription-polymerase chain reaction (RT-PCR)

Total RNA was isolated with TRIzol Reagent (Invitrogen, Carlsbad, California, USA) and quantified by spectrophotometry at 260 nm. On a GeneAmpH PCR System 9700 thermocycler (Applied Biosystems, Foster City, CA, USA), 2 mg of each total RNA was reverse transcribed with SuperScript III (Invitrogen) at 55°C for 1 hour into total complementary DNA, which was used as the template for the subsequent PCR reactions and analysis. The PCR reactions involved an initial denaturation at 94°C for 5 minutes, followed by 25 to 30 cycles at 94°C for 30 seconds, exposure to an appropriate annealing temperature (58–62°C) for 30 seconds, and then a final incubation at 72C for 45 seconds. The PCR primers for analysis of mRNA were listed in Table S3: GAPDH, OCT-4, and Nanog, CD133. Amplified RT-PCR products were then analyzed on 1% agarose gels and visualized using ethidium bromide staining and a camera system (Transilluminator/SPOT; Diagnostic Instruments, Sterling Heights, MI, USA). The gel images of the RT–PCR products were directly scanned (ONEDscan 1-D Gel Analysis Software; Scanalytic Inc. Fairfax, VA, USA), and the relative densities were obtained by determining the ratio of the signal intensity to the GAPDH band. Gene expression between the test (cyclosporine A treated) and the control groups was compared.

### Ethics statement

A total of 28 human samples were obtained for this study. Samples used for analysis in the laboratory were de-identified and not linked with any personal health information. All parts of this study were approved by the Tri-Service General Hospital Institutional Review Board.

### Statistical analysis

All calculations including tables and figures were done using the statistical software, MedCalc (version 12.3., Mariakerke, Belgium) for analysis. Tables 1 and 2 were recorded using descriptive calculation. Wilcoxon scores (rank sums) test and Chi-square test with Fisher's exact test were used in Tables 3 and 4 for continuous and categorical variables respectively. Multiple logistic regressions with stepwise selection were used in Table 5 to predicate the association of interests, as well as the adjustment of confounders. Figure 4 was measured using linear regression analysis. The receiver operating characteristic (ROC) curve was created by following the regression result for significant predicator, OCT 4, to predict clinical metastasis shown in Figure 5. In addition, the Kaplan-Meier curve was likewise applied for survival analysis shown in Figure 6.

## Supporting Information

Figure S1 **Fluids were extracted from the pleural cavity in patients with MPE.** (A) Samples were prepared via a series of centrifugation in Percoll reagent and cell pellets were suspended in a Percoll density gradient, and finally isolated as the white ring layer (arrows). (B) Cell clusters in primary culture of three representative clusters cases (Cases No.1, 7 and 8) from the patients with MPE of MPE demonstrated morphological heterogeneity varying is sizes with spheroid or grape-like morphology. (Magnification, 100×).
(TIF)

Figure S2 **Diagrammatic illustration of a model of MPE dividing into five sequential steps in metastatic cascade.** (1) Pulmonary adenocarcinoma cells composed of a subpopulation of cells within the tumor proper bearing the potential properties of CSC called “stationary or dormant CSCs” and characteristics of EMT undergo direct invasion through the visceral pleura, subsequently enter the pleural cavity leading to malignant effusions. (2) Isolated or small clusters of cancer cells in effusions under the threat of “anoikis” may not only survive; but can also proliferate without limit due to the unique microenvironment of effusions. (3) This special premetastatic niche contains abundant growth factors, chemokines and cytokines, secreted by inflammatory cells, and/or possibly mesothelial cells, which can supply the cancer tissue through autocrine and/or paracrine interactions. (4) Following effusion, which provides nutrients as a dynamic reservoir, CSCs with the capabilities of self-renewal and tumor initiation may grow rapidly and form spheroids or larger cell clusters. (5) Some cells within the spheroids may activate via signaling from the microenvironment again stimulate the characteristics of EMT and properties of CSC becoming “migratory or metastatic CSCs”, further break through the adjacent parietal pleura and invade the angiolymphatic vessels, which provide a route for further migration to the distal organs.
(TIF)

Table S1 **The original data including gender, age, TNM, and immunoexpression of three CSC-representative markers in 20 patients with MPE.**
(DOCX)

Table S2 **Three groups of representative primary antibodies chosen for identification: pulmonary adenocarcinoma-confirming markers, EMT-associated markers and CSC-representative markers.**
(DOCX)

Table S3 **Oligonucleotide sequence of primers used for RT-PCR.**
(DOCX)
