# Supplementary figures and images for: Pulmonary Adenocarcinoma in Malignant Pleural Effusion Enriches Cancer Stem Cell Properties during Metastatic Cascade
Source: PLoS One. 2013 May 1;8(5):e54659. doi: 10.1371/journal.pone.0054659 (PMC3641054; doi:10.1371/journal.pone.0054659)

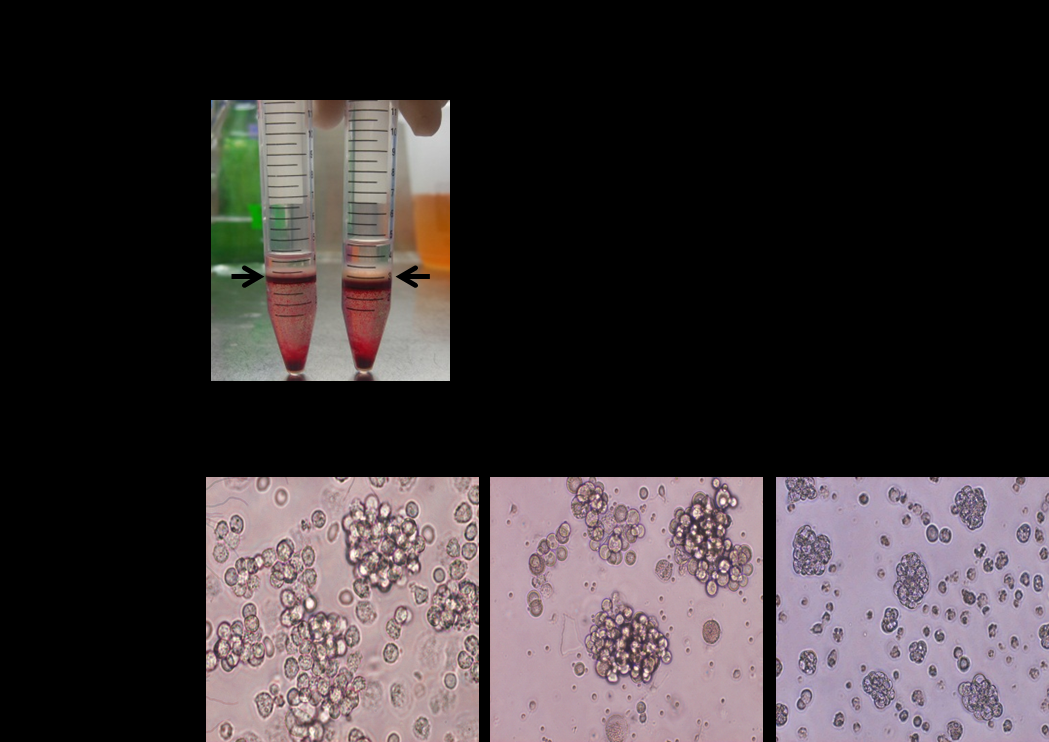

Supplement: Figure S1 — Fluids were extracted from the pleural cavity in patients with MPE. (A) Samples were prepared via a series of centrifugation in Percoll reagent and cell pellets were suspended in a Percoll density gradient, and finally isolated as the white ring layer (arrows). (B) Cell clusters in primary culture of three representative clusters cases (Cases No.1, 7 and 8) from the patients with MPE of MPE demonstrated morphological heterogeneity varying is sizes with spheroid or grape-like morphology. (Magnification, 100×). (TIF) [file pone.0054659.s001.tif]

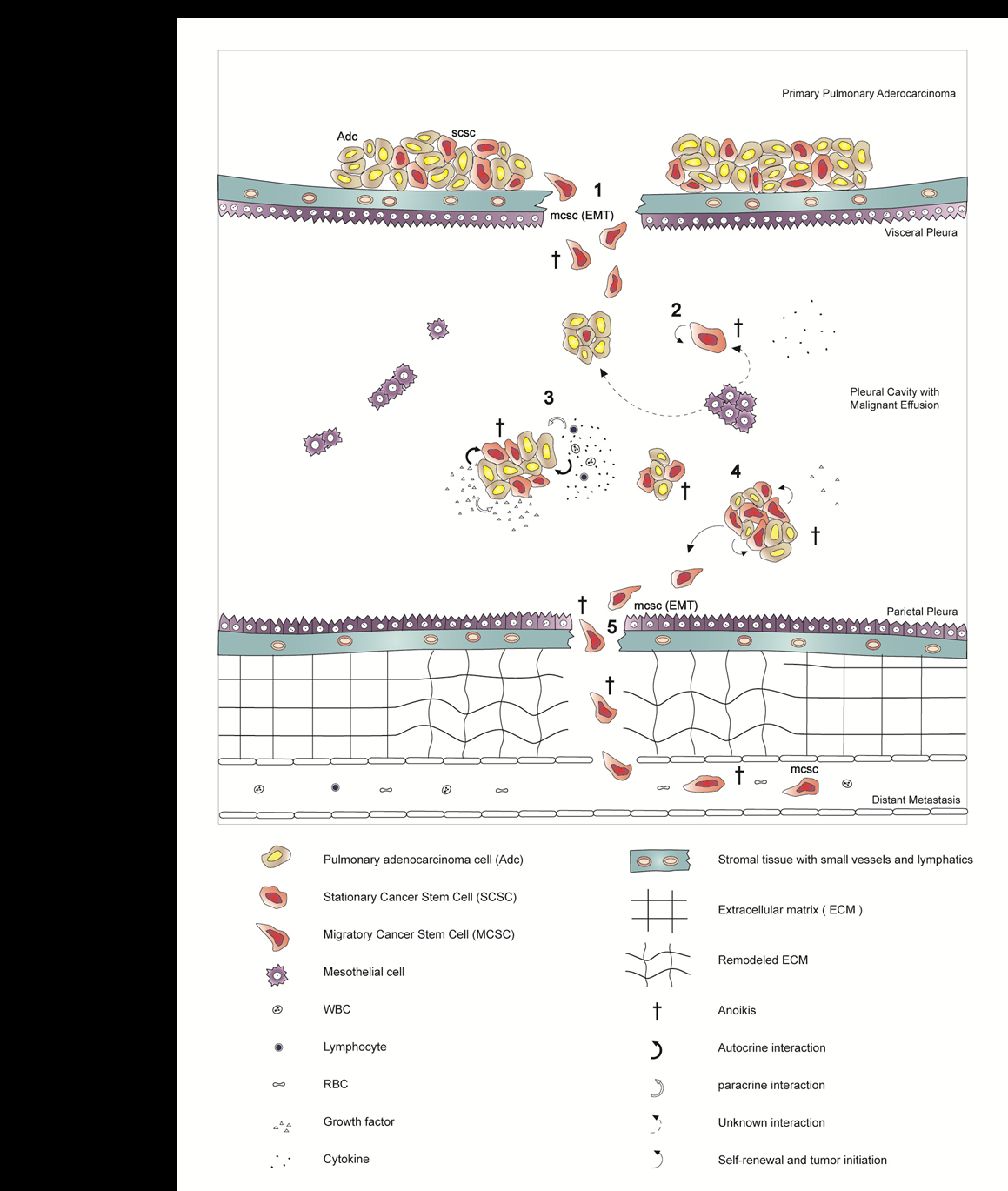

Supplement: Figure S2 — Diagrammatic illustration of a model of MPE dividing into five sequential steps in metastatic cascade. (1) Pulmonary adenocarcinoma cells composed of a subpopulation of cells within the tumor proper bearing the potential properties of CSC called “stationary or dormant CSCs” and characteristics of EMT undergo direct invasion through the visceral pleura, subsequently enter the pleural cavity leading to malignant effusions. (2) Isolated or small clusters of cancer cells in effusions under the threat of “anoikis” may not only survive; but can also proliferate without limit due to the unique microenvironment of effusions. (3) This special premetastatic niche contains abundant growth factors, chemokines and cytokines, secreted by inflammatory cells, and/or possibly mesothelial cells, which can supply the cancer tissue through autocrine and/or paracrine interactions. (4) Following effusion, which provides nutrients as a dynamic reservoir, CSCs with the capabilities of self-renewal and tumor initiation may grow rapidly and form spheroids or larger cell clusters. (5) Some cells within the spheroids may activate via signaling from the microenvironment again stimulate the characteristics of EMT and properties of CSC becoming “migratory or metastatic CSCs”, further break through the adjacent parietal pleura and invade the angiolymphatic vessels, which provide a route for further migration to the distal organs. (TIF) [file pone.0054659.s002.tif]
