# Supplementary material for: Pulmonary Adenocarcinoma in Malignant Pleural Effusion Enriches Cancer Stem Cell Properties during Metastatic Cascade
Source: PLoS One. 2013 May 1;8(5):e54659. doi: 10.1371/journal.pone.0054659 (PMC3641054; doi:10.1371/journal.pone.0054659)

**Table S1.** The original data including gender, age, TNM, and immunoexpression of three CSC-representative markers in 20 patients with MPE.


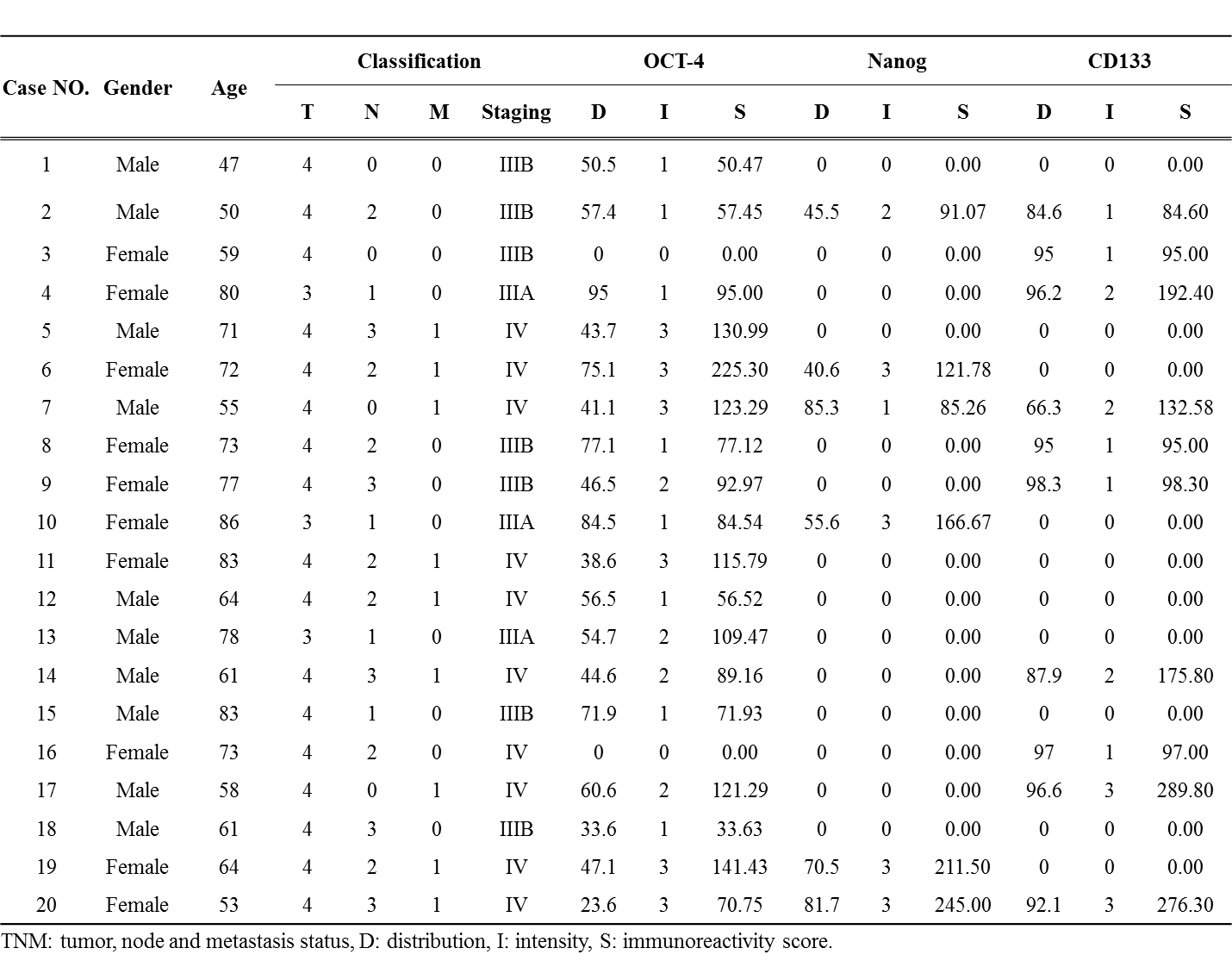

Supplement: Table S1 — The original data including gender, age, TNM, and immunoexpression of three CSC-representative markers in 20 patients with MPE. (DOCX) [file pone.0054659.s003.docx]
