# Supplementary material for: Pulmonary Adenocarcinoma in Malignant Pleural Effusion Enriches Cancer Stem Cell Properties during Metastatic Cascade
Source: PLoS One. 2013 May 1;8(5):e54659. doi: 10.1371/journal.pone.0054659 (PMC3641054; doi:10.1371/journal.pone.0054659)
Supplement: Table S2 — Three groups of representative primary antibodies chosen for identification: pulmonary adenocarcinoma-confirming markers, EMT-associated markers and CSC-representative markers. (DOCX) [file pone.0054659.s004.docx]

**Table S2.** Three groups of representative primary antibodies chosen for identification: pulmonary adenocarcinoma-confirming markers, EMT-associated markers and CSC-representative markers.

|  |  | | | |  | |
| --- | --- | --- | --- | --- | --- | --- |
| **Three groups of primary antibodies** | **Antibody Antibody subtype Staining location Dilution Manufacturer** | | | | | |
|  | CK7 | mouse monoclone | cytoplasm | 1:100 | | Neomarker |
| Pulmonary Adc-confirming markers | TTF-1 | mouse monoclone | nucleus | 1:100 | | Neomarker |
|  | CEA | rabbit polyclone | nucleus | 1:5000 | | Neomarker |
|  | E-cad | rabbit polyclone | membrane | 1:200 | | Santa Cruz |
| EMT-associated markers | Vimentin | mouse monoclone | cytoplasm | 1:100 | | DAKO |
|  | Fascin | mouse monoclone | cytoplasm | 1:200 | | Thermo |
|  | CD133 | rabbit polyclone | membrane | 1:500 | | Abcam |
| CSC-representative markers | Nanog | rabbit polyclone | nucleus | 1:50 | | Abcam |
|  | OCT-4 | rabbit polyclone | nucleus | 1:250 | | Abcam |
| Adc: adenocarcinoma, CK7: cytokeratin 7, TTF-1: Thyroid Transcription Factor-1, CEA: Carcinoembryonic Antigen, E-cad : E-cadherin. | | | | | | |
