# Supplementary material for: Pulmonary Adenocarcinoma in Malignant Pleural Effusion Enriches Cancer Stem Cell Properties during Metastatic Cascade
Source: PLoS One. 2013 May 1;8(5):e54659. doi: 10.1371/journal.pone.0054659 (PMC3641054; doi:10.1371/journal.pone.0054659)
Supplement: Table S3 — Oligonucleotide sequence of primers used for RT-PCR. (DOCX) [file pone.0054659.s005.docx]

**Table S3. Oligonucleotide sequence of primers used for RT-PCR**

| Name  (Accession No.) | Primer sequence | Fragment size  (bp) |
| --- | --- | --- |
| OCT4  (NM000684) | sense: CGCACCACTGGCATTGTCAT | 206 |
|  | antisense: TTCTCCTTGATGTCACGCAC |  |
| Nanog  (NM000024) | sense: AATACCTCAGCCTCCAGCAGATG | 146 |
|  | antisense: CTGCGTCACACCATTGCTATTCT |  |
| CD133  (BC013734) | sense: TCCACAGAAATTTACCTACATTGG | 77 |
|  | antisense: CAGCAGAGAGCAGATGACCA |  |
| GAPDH  (BC001301) | sense: AGCCGCATCTTCTTTTGCGTC | 816 |
|  | antisense: TCATATTTGGCAGGTTTTTCT |  |
